# Capability of Children with Hearing Devices: A Mixed Methods Study

**DOI:** 10.1093/deafed/enad010

**Published:** 2023-05-02

**Authors:** Wouter J Rijke, Anneke M Vermeulen, Christina Willeboer, Harry E T Knoors, Margreet C Langereis, Gert Jan van der Wilt

**Affiliations:** Department for Health Evidence, Radboud University Medical Centre, Nijmegen, The Netherlands; Audiologisch Centrum, Royal Dutch Kentalis, The Netherlands; Department of Research, Pento, Speech and Hearing Centers, The Netherlands; Behavioural Science Institute, Radboud University, Nijmegen, The Netherlands; Audiologisch Centrum, Royal Dutch Kentalis, The Netherlands; Behavioural Science Institute, Radboud University, Nijmegen, The Netherlands; Department of Research, Pento, Speech and Hearing Centers, The Netherlands; Department for Health Evidence, Radboud University Medical Centre, Nijmegen, The Netherlands

## Abstract

We investigated 34 deaf and hard-of-hearing children with hearing devices aged 8–12 years and 30 typical hearing peers. We used the capability approach to assess well-being in both groups through interviews. Capability is “the real freedom people have to do and to be what they have reason to value.” Speech perception, phonology, and receptive vocabulary data of the deaf and hard-of-hearing children, that were used retrospectively, showed a large variability. The analysis of the relation between clinical quantitative outcome measures and qualitative capability interview outcomes suggests that at this age, differences in clinical performance do not appear to translate into considerable differences in capability, including capability did offer insight into the factors that appeared to ensure this equivalence of capability. We argue that capability outcomes should be used to determine the focus of (auditory) rehabilitation and support, in line with the United Nations Convention on the Rights of the Child.

## Introduction

Optimization of patients’ functioning and enabling their participation in valued activities are core elements of rehabilitation. In fact, it is a nation’s responsibility that persons with disabilities can exercise their right to make decisions for their lives and be active members of society (*Convention on the Rights of Persons with Disabilities*, 2006). To evaluate efforts that aim to contribute to this goal, there should be an account of freedom to make decisions, in addition to insights into what activities are considered valuable to individuals and the society of which they are a part. Societies are groups of people who live together in specific ways. Not living together is hardly an option for human beings, but how they live together is subject to a considerable degree of variation. Living together confers benefits to individual members, but some seem to succeed in gaining more benefit out of it than others. A continual question for societies is how such relative advantages and disadvantages should be assessed and what, if anything, should be done to mitigate them (Wolff & De-Shalit, 2007). There is no undisputed answer to this long-standing question. According to some, the increase in the aggregate surplus of pleasure over pain is the single appropriate yardstick (Lazari-Radek & Singer, 2014). However, such utilitarian views have been fiercely criticized (e.g., Rawls, 1999). Building on the work by Rawls, Nobel laureate Amartya Sen has suggested that relevant differences in advantages and disadvantages among people can best be captured in terms of their capability (Sen, 2009). In the capability approach, individual advantage or disadvantage is judged by a person’s capability to do things he or she has reason to value; the focus is on the freedom that a person actually has to do this or be that (Sen, 2009, p. 231). The capability approach offers a specific informational focus in judging and comparing overall individual advantages (Sen, 2009, p. 232). As such, it proposes a specific way of answering questions like: Are, in a specific society or community, individuals with a particular disability disadvantaged as compared with their non-such-disabled peers? Specifically, it invites us to address such question by probing into the freedom that such persons have to do or be things they have reason to value.

**Figure 1:**
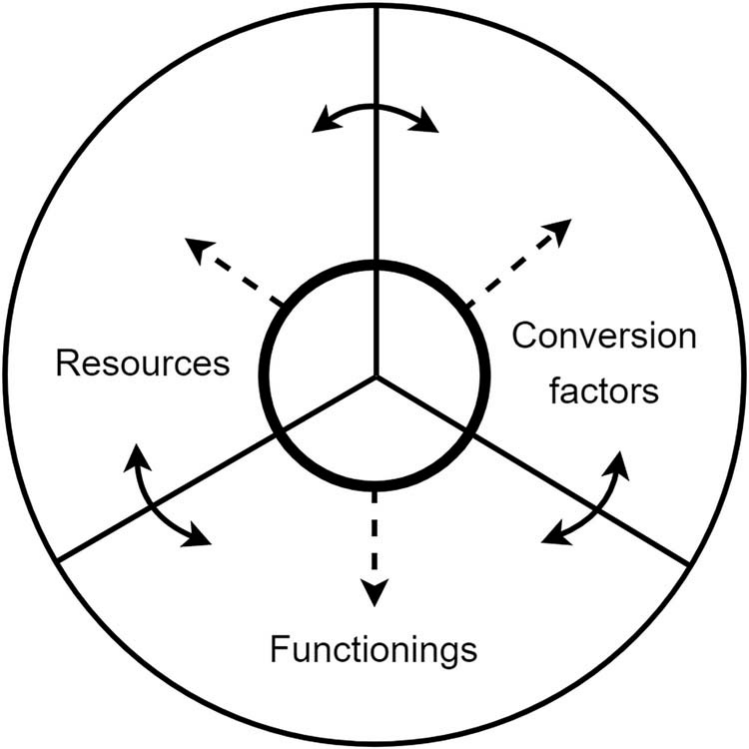
The concept of capability. Capability is the set of real opportunities that people have to be and to what they have reason to value. Capability is an interplay of realised opportunities (called functionings), the necessary resources and personal, social, and environmental factors (called conversion factors).

The capability approach is not a measure of self-reported (health-related) quality of life. The quality of life of deaf and hard-of-hearing (DHH) children and typical hearing children is often found to be similar (Loy et al., 2010; Meserole et al., 2014; Razafimahefa-Raoelina et al., 2016; Warner-Czyz et al., 2009), whereas it would be unlikely to assume they are similar in their advantages and disadvantages. Sen developed the capability concept explicitly as a metric for expressing the relative advantages and disadvantages that people have. He held that in this respect, the real opportunities that people have to do and be things they have reason to value is more relevant than subjective wellbeing or possession of primary goods (Sen, 2009). He also emphasized that capability is the outcome of the interplay between resources, conversion factors, and functionings (“doings and beings”), visualized in Figure 1.

Resources can be conceived as production factors, such as hearing devices, Hearing Assistive Technologies, or even money. Conversion factors are all personal, social, and environmental factors that determine whether people can actually deploy those resources in such a way as to achieve something of value. For example, a personal conversion factor that determines the value of a hearing device is the experienced gain/benefit of it. Social conversion factors can be acceptance and understanding from others, whereas environmental conversion factors might be related to acoustics. Functionings are the things that people then actually do and are, such as hearing another person, speaking, reading, or socializing.

It could be argued that rehabilitation should result in strengthening recipients’ capability. The question is, however, what counts as evidence of enhanced capability, and how such evidence can best be obtained. Capability enables people to freely choose to do (e.g., to meet with friends) and be (e.g., healthy, independent) anything they have reason to value. Hence, adopting the capability approach in (auditory) rehabilitation and support would require two things:

- A reflection on the nature of the valued modes of doing and being in a particular context: What may be considered of such general value that it should, at least to some extent, be attainable for all members of that community?
- An analysis of the conditions of opportunity (resources, conversion factors and functionings, and their interplay).

Although the capability concept appears quite congenial to the theory and practice of rehabilitation, its application to the field is still in its infancy. In the recent years, there have been some excellent reviews of several capability measurement instruments (Proud et al., 2019; Ubels et al., 2022) and their use in economic evaluations (Helter et al., 2019). These reviews show how capability can capture the outcomes of value in health and economic evaluations. However, they also conclude that these instruments fail to reflect the burdens people experience to achieve capability, whereas also unable to include the participants’ contexts. We noticed the same limitations in a previous study, measuring capability in deaf children with cochlear implants through a questionnaire (Rijke et al., 2019).

The objective of this study was to observe and study DHH children through a capability lens. Especially for (auditory) rehabilitation purposes, it would be relevant to know how the capability approach can be of added value.

## Materials and Methods

### Participants

During the inclusion period of the study (12 months), we included 64 children between the age of 8 and 12 years old that attended primary education: 34 DHH children and 30 typical hearing children. DHH children all used unilateral or bilateral hearing devices: 23 had cochlear implants, and 11 had hearing aids. The hearing aid children had hearing losses starting at 25 dB (average thresholds at 1, 2, and 4 kHz). DHH children were approached to enroll in the study before their annual fitting of their hearing device and follow-up evaluation of their development in their out-patient clinic. We excluded non-Dutch speaking children or children with additional severe complex needs, who were not able to express themselves in an interview setting. We did not preselect for other factors. We included typical hearing children to explore what interests, functionings, resources, and conversion factors are distinct for DHH children, and what rehabilitation should provide for them to maximize their capability in a society focused primarily on typical hearing people. Mainstream primary schools were approached for the inclusion of typical hearing children, who were interviewed in groups of five. It was not practical nor ethically warranted to invite typical hearing children to the hospital to ensure the same interviewing context as DHH children.

### Assessments

To determine capability, information on interests, resources, conversion factors, and functionings was collected both qualitatively and quantitatively in DHH children. We included standard auditory and psycholinguistic assessments: Speech perception, receptive spoken language vocabulary, verbal working memory, and phonological processing. These assessments were included to explore how potential differences in the qualitative outcomes could be explained by poor or high scores on speech and auditory tests. The clinical measures concern the mechanisms of construction, maintenance and processing, and the retrieval of meaning of phonological memory traces that are involved in communication in spoken language (Nittrouer et al., 2013; Pisoni et al., 2011). Verbal working memory and phonological processing are a prerequisite for language development. Both typical hearing children and DHH children were interviewed; the clinical indicators were only assessed in the latter.

### Capability Interview

All participating children and their parents were informed that the overall goal of the interview was to learn more about how they are doing, what is important in their daily lives, and what factors are helping or hindering them in doing or being what they think is valuable. The interviews with the DHH children were carried out after the regular check-up of their hearing devices, to ascertain optimal speech perception during the interview. Children could choose whether a parent was present during the interview. No interviews were interrupted, aside from one interview with typical hearing children; this was shortly halted due to outside construction noises. Interviews were fully audio recorded, and intelligent (non-verbatim) transcripts were subsequently created. The children had not met the interviewer (the first author) previously. The interviewer (male, late-twenties, typical hearing) had a background in psychology and was trained in qualitative research. The interviews were in spoken Dutch. Prior to the interviews, the child’s need for a sign language interpreter was inquired with the parents but was waived for all children. One child attended a school where sign supported speech was used. He said that unless there was loud music, he could hear and understand the interviewer.

The main questions in the interview were centered to daily activities: “*Would you tell me something about a typical day, and a typical week? What do you do, where do you go, and what do you need to do those things?*” These questions were used to stimulate the child to formulate as much as possible without priming from the interviewer. When describing a typical week, themes like school, leisure activities, sports, social events, and family time came up. To ensure that we systematically captured as broad a range of themes as possible, we framed the interview with the seven basic goods proposed by John Finnis (1980): Life, knowledge, play, sociability, practical reasonableness, aesthetic experience, and transcendence. The interviewer ensured these were addressed in the interviews. Answers were typically met with questions to elaborate (i.e., “*How?*”, “*Why?*”, “*Can you tell me more about that?*”) and questions about the child’s choices, reasoning, resources, support, and obstacles, such as “*Are there things that you would like to do or be, that you are not capable of right now?*” and “*What do you use most to achieve that?*”

### Speech Perception

Speech perception abilities were assessed with the NVA Dutch open set identification test, containing meaningful consonant–vowel–consonant words (Bosman & Smoorenburg, 1995). Stimuli were presented in a sound-treated booth at a presentation level of 65 and 45 dB SPL in quiet, and subsequently at 65 dB SPL with a 65 dB SPL noise level, resulting in a 0 dB speech/noise ratio. Words and noise originated in the same loudspeaker, with continuous noise with spectral speech characteristics. Stimuli were presented via loudspeakers, where no speech reading was possible. Response was given by repetitions of the perceived item. Speech perception was quantified as the percentage phonemes correctly repeated. This resulted in three scores: Speech perception at 65, 45, and 65 dB in noise.

### Receptive Spoken Language Vocabulary

Receptive spoken language vocabulary was assessed with the Dutch version of the Peabody Picture Vocabulary Test-III-NL (PPVT) (Dunn & Leota, 2005). The PPVT can be assessed in people aged 2 through 90. Stimuli consisted of words that were presented live, and speechreading was possible. The task consisted of identifying the stimulus-word out of four pictures. Spoken language vocabulary was expressed in one score, the word quotient, which is calculated based on age norms. The average quotient is 100 with an average standard deviation of 15.

### Verbal Working Memory

Verbal working memory was assessed with two subtests of the Clinical Evaluation of Language Fundamentals 4: Digit span forward and digit span backward. In these tasks, spoken digit strings of increasing lengths must be repeated forward or backward (Semel et al., 2003). Forward repetition reflects the ability to store information in the phonological loop of the verbal working memory. Backward repetition appeals on the slightly different ability to process information in verbal working memory. Verbal working memory is therefore expressed in two scores, where the average norm score is 10 with a standard deviation of 3.

### Phonological Processing

A non-word repetition task was used to assess phonological processing. With no speech reading possible, 16 Dutch non-words had to be repeated, for example/ji’nus/. This task focusses on measuring the encoding skills and phonological storage. The words consist of two to five syllables. Phonological processing was expressed in a single score, the correct percentage of repeated phonemes. On average, school-going children with TH score 92.4%, with a standard deviation of 2.9% (Bree et al., 2007).

### Analyses

Qualitative data were analyzed using a deductive (or directed) qualitative content analysis (Mayring, 2000). This type of content analysis has the researcher starting with predetermined codes from an existing theory, which in this case is the capability approach. Excerpts were coded as either interest, resource, conversion factor, or functioning, following the central elements in the capability approach. In the coding process, interests are what a child identifies as important, fun, or desirable. Resources are the materials and means that children depend on to realize their interests. Conversion factors are the personal, environmental, and social factors that influence how resources lead to capability. Functionings are the valuable things that children actually do and be. These are often dependent on resources and conversion factors. Codes could overlap, as an excerpt could contain information on more than one code (e.g., a cochlear implant can be both a resource as a conversion factor, depending on the interest). The coding process was computer assisted, using ATLAS.ti version 8 for Windows. Every answer was selected and coded to fit the closest description of an interest, resources, conversion factor, or functioning. The interrater reliability analysis, represented by Kappa, was performed in six interviews with another PhD student to determine consistency among raters (Giacomini & Cook, 2000; MacPhai et al., 2016). After coding, documents were generated by ATLAS.ti with codes per group. For example, a document could hold all resources (code) for typical hearing children (group). These were then listed in tables that are discussed in the Results section.

The following quantitative clinical indicators were used to support our qualitative analysis: Speech perception in quiet at 65 and 45 dB, in noise at 65 dB SPL SN = 0, receptive vocabulary, digit span forward and backward, and phonological processing. To explore how results from clinical indicators relate to interview outcomes, we selected children who performed either poorly (in the lowest quartile) or highly (in the highest quartile) on three or more of the seven clinical indicators and explored their interview outcomes. Potential differences in clinical indicators between children with hearing aids or cochlear implants are beyond the scope of this paper and will therefore not be reported.

## Results

The following results describe differences between DHH children and typical hearing children. The interrater reliability was .84, which is considered high. Table 1 lists the demographic characteristics.

**Table 1:** Demographic characteristics of the children with cochlear implants, hearing aids, and typical hearing children

Characteristic		Children with cochlear implant(s)	Children with hearing aid(s)	Typical hearing children
*n*		23	11	30
Age in years	M (SD)^a^ Range	10.1 (1.2) 7.8–12.0	9.2 (.6) 8.3–10.4	11.1 (1.0) 9.0–12.0
Age of first aid in years	M (SD) Range	3.4 (2.3) 1.1–8.1	3.7 (2.4) .1–6.4	Not applicable Not applicable
Gender	M F	16 7	5 6	12 18
Aids^a^	Unilateral Bilateral	7 16	0 11	Not applicable
Education^a^	Mainstream Special	13 10	11 0	30 0

^a^ *Significant difference between groups*

Groups were statistically different in age (*H* (2) = 11.188, *p* = .004), unilateral or bilateral fitting (χ^2^ (1) = 4.213, *p* = .04), and mainstream or special education (*χ*^2^(2) = 21.127, *p* < .001).

### Clinical Indicators

**Figure 2:**
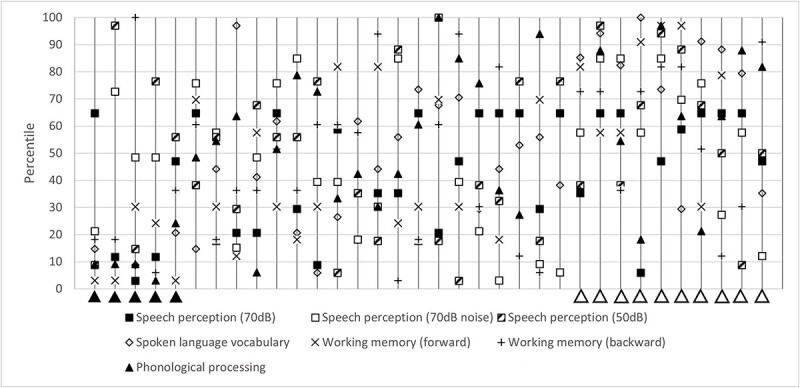
Performance of 34 deaf and hard-of-hearing children on clinical indicators in percentiles. Every vertical line is a DHH respondent. Poor and high performers are marked with solid and transparent triangles, respectively.

The majority of the participated DHH children perform adequate on the clinical indicators. Five children scored in the lowest quartile of at least three of the seven clinical indicators. To explore the interviews supported by the clinical indicators, we calculated the percentile of every clinical indicator for every DHH child. We displayed these percentiles in Figure 2, where every vertical line is a DHH child, in order of poor performance to high performance.

The outcomes of the five children who performed poorly on the clinical indicators are interesting for further investigation. What are their characteristics and what do they say in the interviews on their capability? All five scored in the lowest quartile of spoken language vocabulary and phonological processing. They were all boys with a cochlear implant in special education. In contrast, 10 of 34 DHH children scored in the highest quartile of at least 3 of the 7 clinical indicators. They were six girls, four boys, all attending mainstream education. Seven of the 10 had cochlear implants. Their interview outcomes are discussed further in the Results section, under *Interview outcomes from children with poor or high performance on clinical indicators*.

### Functionings, Resources, and Conversion Factors

When talking about their daily lives, DHH children mentioned similar “doings and beings” as typical hearing children. Children of both groups went to school, with some children with cochlear implants being enrolled in special education. Both DHH and typical hearing children cycled to go to school, sports, or other activities. However, children attending special education did rely on their parents to give them a ride to school and to other activities. All children participated in Physical Education in school, liked to play outside, and most children were a member of a sports club. However, although DHH children tended to engage in similar daily activities (functionings) as their hearing peers did, they were dependent on supporting resources and conversion factors to be able to do so. These functionings, resources, and conversion factors are listed in Table 2. When these supporting resources and conversion factors were absent, they acted as barriers for capability.

**Table 2:** Functionings of typical hearing children compared with functionings of deaf and hard-of-hearing children, and related resources and conversion factors

Typical hearing	Deaf and hard-of-hearing		
Functionings	Functionings	Resources	Conversion factors
General, i.e., not related to specific functionings	=	Cochlear implant or hearing aid	Full batteries, big earpieces
		Hearing Assistive Technologies	Correct use by wearer, acceptance of peers, feel different
		Flashing light doorbell Vibrating alarm clock	Uncomfortable sounds
			Dependence on lip reading
			Feel dependence on hearing device
			More assertiveness is needed to get others to help
			Parents are afraid hearing device could get lost, broken
			Loud noises lead to high beeps
School	=	Hearing Assistive Technologies	Acoustics, direction of sound, echo in Physical Education class, acceptance and understanding from peers, teachers
		Peripatetic teaching	
		Special education (smaller classrooms, adapted acoustics, teachers capable of sign language)	Taxi, additional support
Sports	=	Accessories for hearing device and Hearing Assistive Technologies: (swimming) caps, cords, headbands, (water) cases, clips, cables.	Parents: play a role in choosing sport or safety measures.
Tennis, gymnastics, ballet, hockey, competitive swimming, korfball, soccer	= Ice skating, volleyball, horseback riding, kickboxing		Vulnerability of hearing devices: could fall off, is not waterproof, could take a hit. Swimming interpreter.
	No sailing		Hearing device could get lost
Riding the bike, both alone, together, talking	=		Help from others in traffic, some have trouble identifying direction of sound, hearing cars.
Swimming	Not hearing others while swimming	Water case	Lip reading, supporting signs, sign language, having to dry ears and hair before being able to put on hearing device
Play outside	=		Sweater with cap to protect cochlear implants
Spend time with friends	=		Late home from school (when attending special education)
Holiday	=		Not being able to fly due to cabin pressure
Trampoline	=		Headpiece falls of when jumping
Watching TV/Netflix/YouTube		Hearing Assistive Technologies	Subtitles, direct audio streaming
Music listening, making	=	Hearing Assistive Technologies	Direct audio streaming
Sleepovers	=	Charger	

*Note.* An equal sign (=) depicts similar functionings compared with typical hearing children

Other functionings that were mentioned by both typical hearing children and DHH children were spending time with family, playing games, playing on the playground, drawing and crafting, reading, playing with LEGO sets, and singing along with music. In conversations with DHH children, parental involvement was a frequent topic compared with typical hearing children. Parental engagement was mentioned as companionship or support and guidance when deciding what extracurricular activities are appropriate.

“*My best friend is… that big guy there! [points to father]. In school I have friends, but not best friends.*”*—boy, 8, cochlear implants, mainstream education.*

“*I always wear my hearing aids. Except with sleeping, showering, and swimming. And sailing because my parents were nervous about that. Because I could lose one.*”*—boy, 9, hearing aids, mainstream education.*

During analysis, it stood out that although there was little difference in the number of children that engaged in activities related to music (17 of 30 typical hearing children compared with 17 of 34 DHH children), there was a difference in the kind of activities. In typical hearing children who engaged in music-related activities, 11 of 17 children played an instrument, compared with 7 of 17 DHH children, where the majority preferred to sing and dance. Two typical hearing children mentioned that they participated in a musical or theater production.

### Interests

Interests represent what children identified as important, fun, or desirable. Table 3 compares interests of typical hearing children and DHH children.

**Table 3:** Interests of typical hearing children compared with deaf and hard-of-hearing children

Typical hearing	Deaf and hard-of-hearing children
Physical education, love hate relationship with school, sports	=
Listening to music	=
Singing	=
Dancing	=
Playing an instrument	=
Musical	
Theatre (acting)	
	Be able to hear people or media
	Be able to be normal
	Others not to notice their deafness
	Others to be aware of what it’s like to have hearing loss
	Feel like not being able to become a teacher, because of her hearing loss
	Be able to hear traffic, direction, and chat while cycling
	Be able to shower with sounds
	Be able to use sign language when hearing is not possible
	Be able to turn off their hearing devices to have silence, when sleeping or fighting
	Be able to play sports without restrictions of their hearing loss, use of their hearing devices, assistive equipment, or protective gear for their device. Be able to hear whistles of referees.
	Be able to swim and still hear, without the need for waterproof housings.

*Note.* An equal sign (=) depicts similar functionings compared with typical hearing children

DHH children frequently mentioned situations in which they would like to be able to hear people or media (videos and music). Children reported that they would like to be “normal” (boy, 9, cochlear implants, mainstream education), other people to not notice their deafness (boy, 9, hearing aids, mainstream education), and they would like other people to be aware of what it is like to have a hearing loss (girl, 8, hearing aids, mainstream education). One girl felt like she would not be able to become a teacher, because of her hearing loss:

“*I like a lot of professions for later, but I can’t decide yet. And some are off for me, because I can’t do it with hearing aids, like a teacher or something like that.*”*—girl, 9, hearing aids, mainstream education.*

Most children liked sports. DHH children did mention challenges during sports that typical hearing children did not. They would have liked to play sports without the restrictions that come along with their hearing loss or the use of their hearing devices or assistive equipment. Their comments included they would like to be able to detect the sound of whistles of referees and they do not like wearing protective gear for the device. These children never brought up quitting their sport despite these restrictions, however.

*“I’m not allowed to only wear just a cap [when ice-skating], because my mother’s afraid the cochlear implant will break if I fall. So, I must wear a helmet. […] it’s a shame, because I’m now in the competition team, and it’s about hundredths and thousands and that helmet…”—Girl, 11, cochlear implants, mainstream education.*

“*On sailing camp, I didn’t wear my hearing aids because my mother said I could lose one.*”*—Boy, 9, hearing aids, mainstream education.*

### Interview outcomes from children with poor or high performance on clinical indicators

The interview responses from DHH children who performed poorly on clinical indicators showed that they did not have many friends and preferred to engage in activities alone. Children who scored in the lowest quartile in only two of the seven indicators did not express statements such as engaging in activities alone or not having many friends. One boy mentioned in the interview that he sometimes has trouble distinguishing voices amongst people. He used Hearing Assistive Technologies in school only. He also played sports, liked gaming, and playing with LEGO sets. Another boy mentioned having a very close relationship with his father, saying he is his best friend. He played tennis and liked to play volleyball. His biggest issue with his cochlear implants was that he cannot use them while swimming.

Relatively high-performing children did not report experiencing major problems in their daily lives, and their daily use of hearing aids or cochlear implants ranged from wearing it only to school to wearing it all day, every day, and using Hearing Assistive Technologies and waterproof cases. Although major problems in functioning are averted, these 10 children did include the girl who cannot reach top speeds in ice-skating due to the helmet she wears for her cochlear implants and a girl that was not allowed to wear her cochlear implant when sailing with friends, as she could lose it. In addition, these 10 collectively report difficulties in busy classrooms, hearing direction in traffic, and batteries being empty in inconvenient moments.

## Discussion

An encouraging key finding of our study was that DHH children barely differed from typical hearing children in terms of self-reported functionings (what they do/are). However, depending on performance on clinical indicators, they appeared to be dependent on specific conversion factors. These included Hearing Assistive Technologies, the management and coping with environmental noise, and the technical features of the devices, such as connectivity with other devices and volume control. For many children, assistive devices are a way of controlling input from their surroundings. How the devices function as conversion factor seems to determine the degree of freedom DHH children experience. Another conversion factor is parental involvement, both for support and for advice when deciding how to use cochlear implants or hearing aids during extracurricular activities. Although the dependency of children on their parents for converting capability into functionings is not surprising (Ballet et al., 2011; Biggeri et al., 2006), it is notable how their parents were mentioned frequently in interviews with DHH children as compared with typical hearing children. This is especially noteworthy as no question in the interviews specifically addressed parental involvement. DHH children described this involvement as both supportive (e.g., an 8-year-old’s father being described as his best friend) and obstructive (e.g., not being allowed to sail). Multiple recent studies made observations that identified parents’ involvement in DHH children as multifaceted (Erbasi et al., 2018), higher among mothers than fathers (Brand et al., 2018), and resulting in parental stress (Zaidman-Zait et al., 2015). Previous studies reported that teachers had considerably lower expectations of DHH children, which resulted in permitting them to take less responsibility (Meinzen-Derr et al., 2018; Reed et al., 2008; Smith, 2008). This is problematic, as it could lead to a state of learned helplessness and a higher dependency on others, such as their parents or teachers (Mathews, 2015; Wolters et al., 2012).

### Being Different

Another notable finding is that both children with hearing aids and cochlear implants express interests not heard in typical hearing children. They report the desire to not differ from their TH peers, to be “normal,” and that others do not notice their deafness. Moreover, they want to be accepted and understood by their peers. At the same time, they want their special needs to be acknowledged. They wish others to understand that communicating in a busy classroom is challenging and tiring, and that they need sign language when spoken language does not suffice, and that they need to ask others to reiterate something they said. Although sign language is often a second language for children with hearing aids and cochlear implants, the wish to be able to communicate at all times should be recognized. The interplay between wanting to be normal but still wanting to be acknowledged is worth noting, as problems may arise when deafness is not fully accepted or coped with at a later age (Castellanos et al., 2018; Wolters, 2013). Also, children that receive additional support, such as special education, attend school further away from home and cycle less, which might have repercussions for social relationships in their neighborhood (Dirks & Knoors, 2019).

Remarkably, whilst it being their most important resource, many of the DHH children noted that the best feature of their hearing device is that it could be turned off. Hearing through a hearing device is known to be effortful and fatiguing, as sounds and speech discrimination can be extremely limited in noisy environments, making fluent communication challenging (Hicks & Tharpe, 2002; Lewis et al., 2016; Ohlenforst et al., 2017). Frequent and prolonged daily use is encouraged, however, as it is correlated with higher speech perception scores (Easwar et al., 2018; Guerzoni & Cuda, 2017).

### Comparing Clinical Indicators and Interview Outcomes

Relatively poor performance on one or two clinical indicators (such as speech perception and phonological processing) did not appear problematic based on interview outcomes. Children tended to compensate on different areas, leading to fewer problems in pursuing valuable activities. When children performed poorly on three or more indicators, compensating seemed more difficult. These five children, all attending special education, talked about situations with less social interactions, and more involvement from their parents. These findings require more in-depth research as to why compensating might be difficult. Explanations could be related to problems in managing social contacts (Antia et al., 2012), less developed language skills (Hall et al., 2019), or perhaps suboptimal development of executive functions (Boerrigter, 2021).

### Limitations and Implications

A limitation of this study is the potential bias in responses from children. Interviews with typical hearing children took place in their own school, compared with the out-patient clinic for DHH children. Typical hearing children all attended the same school, potentially a bias for the choice of activities available in that area. In addition, although group conversations stimulated input, it could have had an impact on openness and honesty. Also, the participating children varied in age and education (as the demographics indicated), but also social contexts and personal histories (as the interviews illustrated). Some studies suggest that potential social challenges develop not at primary school age, but later, during adolescence (Brice & Strauss, 2016; Wolters et al., 2012; Zaidman-Zait & Most, 2020). Impact on capability in different age groups would therefore also be an important follow-up evaluation.

It is important to note that in qualitative research, there is not only the potential bias in responses from children. The personality, background, and perspective of the interviewer might distort responses and follow-up questions. In this case, the interviewer was typical hearing. Although the interview methodology was grounded in theory, it would be interesting for future research to compare interview responses with an interviewer from the Deaf community. In addition, interviews with typical hearing children and DHH children who are classmates could provide insights in the effects of different school environments.

Finally, although Finnis’ basic goods provided a useful framework for the capability interview, it has limitations in its use in children. The seven dimensions (life, knowledge, play, sociability, practical reasonableness, aesthetic experience, and transcendence), relate strongly to adults, which possibly explains why certain subjects (e.g., transcendence) were mentioned only infrequently. These findings therefore cannot be extrapolated to all DHH children, nor were they intended to. Nevertheless, these findings may help us in two specific ways.

First, the added value of measuring capability becomes apparent. The capability approach immediately and logically directs attention to questioning the content of capability: What is it that these children in this context should be able to do. And when constraints for capability appear, the capability model with resources, conversion factors, and functionings leads to identifying action points to rectify these constraints.

Second, the outcomes of the interviews, combined with clinical indicators, underscore particular areas that auditory rehabilitation professionals need to address to enhance the capabilities of DHH children. Auditory rehabilitation is aimed at achieving several objectives, as outlined by the American Speech-Language-Hearing Association (2018), including (a) enhancing the listening pathway through training, (b) facilitating the use of listening technology, (c) fostering language development, (d) compensating for auditory dysfunction through visual access, and (e) providing personalized counseling to individuals and their families. Sen’s capability framework aligns with this in terms of resources (b), functioning (a,c,d), and conversion (d,e). Our study identified crucially important themes that may be considered obstacles to achieving capability including (1) difficulty accessing communication, (2) frustration at the limits of devices, (3) overbearing and dictating parents, (4) lack of peer relationships, (5) correlation to auditory skills/visual access, (6) negative self-perception. All of these obstacles should be addressed in auditory rehabilitation.

Combining speech perception and personalized interviews has high potential to become the preferred approach in the rehabilitation of DHH children, suggests Van der Straaten (2022). Children who have relatively high scores on clinical indicators, generally have good abilities to accomplish their daily activities. Still, they can experience limitations in doing what is valuable to them. Contrarily, children with relatively low clinical scores seem to require help in achieving valuable states. They do not, however, necessarily feel limited to lead valuable lives.

## Conclusion

DHH children who participated in this study often led lives where they, in terms of capability, could do and be things that they have reason to value, not too different from their TH peers. They had friends, played sports, had hobbies, and enjoyed time with family. Considering these children were part of the same society, it is not surprising they strived for similar functionings, a similar norm. In striving to equality in the capability of DHH children and typical hearing children, there are two options: To use the normative level of typical hearing children and identify necessary resources, conversion factors, and functionings, and provide and secure these for DHH children. This option to establish capability seems only reachable for children who obtain adequate clinical indicators. Another option is to strive for equal capabilities compared with those of DHH persons in Deaf Communities. This is to call into question how certain societal norms are defined (Sparrow, 2005). For example, to achieve adequate communication, spoken language might not be the only mode, when sign language is available and culturally accepted. For children with relatively poor clinical indicators, this second option might be preferable. From a capability perspective, speaking and hearing are very valuable functionings leading to many freedoms, but they are a means to an end, which is communication. Sign language, in this example, can be an important valuable functioning, provided that necessary resources and conversion factors are maintained.

We would like to point out that these options for DHH children are described from the perspective of these children themselves. As discussed in the Introduction, however, there is the society’s responsibility to make sure that persons with disabilities can exercise their right to make decisions for their lives and be active members of society. The (change of) behavior toward children with disabilities of *typical hearing* children and adults should therefore also be considered when we aim to improve and protect the capability of DHH children. Typical hearing children and adults should be invited to increase their social interactions with DHH children, which is particularly challenging for DHH children who perform poorly on clinical indicators.

DHH children in this study were more vulnerable than typical hearing children. Although comparable to typical hearing children in functionings, DHH children rely on a range of conversion factors to make sure their hearing tools become enforcers of capability in all aspects of daily life. Their hearing aids or cochlear implants are important resources for all of them: It makes it easier to do or to be things they have reason to value. Other prerequisite, supportive, factors include transportation to their school, Hearing Assistive Technologies to improve adverse listening conditions, and the need for empathy, inclusion, and participation. Furthermore, clinical indicators provide valuable information on auditory gain for speech perception of hearing devices. In addition, collecting information on capability could be a beneficial approach in rehabilitation to improve involvement of the child, by discussing together with parents, and health and education professionals, what they strive for, even if that is outside our direct sphere of influence.

## Data Availability

The datasets presented in this article are not readily available because the interviews contain highly personal and sensitive information, which we do not have the permission to share beyond this manuscript. Requests to access the datasets should be directed to wouter.rijke@radboudumc.nl.
